# Methoxy-Substituted γ-Oxa-ε-Lactones Derived from Flavanones—Comparison of Their Anti-Tumor Activity In Vitro

**DOI:** 10.3390/molecules26206295

**Published:** 2021-10-18

**Authors:** Aleksandra Pawlak, Marta Henklewska, Beatriz Hernández-Suárez, Monika Siepka, Witold Gładkowski, Czesław Wawrzeńczyk, Karolina Motykiewicz-Pers, Bożena Obmińska-Mrukowicz

**Affiliations:** 1 Department of Pharmacology and Toxicology, Wrocław University of Environmental and Life Sciences, C.K. Norwida 31, 50-375 Wrocław, Poland; marta.henklewska@upwr.edu.pl (M.H.); beatriz.hernandez-suarez@upwr.edu.pl (B.H.-S.); karolina.motykiewicz-pers@upwr.edu.pl (K.M.-P.); b.mrukowicz@gmail.com (B.O.-M.); 2 Department of Chemistry, Wrocław University of Environmental and Life Sciences, Norwida 25, 50-375 Wrocław, Poland; monikasiepka@yahoo.com (M.S.); witold.gladkowski@upwr.edu.pl (W.G.); czeslaw.wawrzenczyk@upwr.edu.pl (C.W.)

**Keywords:** flavanones, lactones, flavanone-derived lactones, anti-cancer activity

## Abstract

Background: The study investigated four flavanone-derived γ-oxa-ε-lactones: a parent unsubstituted compound and its three derivatives with the methoxy group in positions 2′, 4′ and 8. Our objective was to find out if the introduction of the methoxy group into the aromatic ring affects in vitro anti-tumor potency of the investigated lactones. Methods: Cytotoxic and pro-apoptotic effects were assessed with cytometric tests with propidium iodide, annexin V, and Western blot techniques. We also investigated potential synergistic potency of the tested lactones and glucocorticoids in canine lymphoma/leukemia cell lines. Results: The tested flavanone-derived lactones showed anti-cancer activity in vitro. Depending on its location, the methoxy group either increased or decreased cytotoxicity of the derivatives as compared with the parent compound. The most potent lactone was the one with the methoxy group at position 4′ of the B ring (compound **3**), and the weakest activity was observed when the group was located at C-8 in the A ring. A combination of the lactones with glucocorticoids confirmed their synergy in anti-tumor activity in vitro. Conclusions: Methoxy-substituted flavanone-derived lactones effectively kill canine lymphoma/leukemia cells in vitro and, thanks to their synergistic action with glucocorticoids, may potentially be applied in the treatment of hematopoietic cancers.

## 1. Introduction

Compounds with a lactone ring in their structure are the subject of numerous studies due to their common occurrence in nature as well as a broad spectrum of biological activity. Lactones are secondary metabolites of various plants, insects, microorganisms and marine organisms and exhibit biologically important properties such as antifeedant, antibacterial, antifungal and cytostatic properties [1]. Lactones bearing an aromatic ring exhibit an exceptionally wide spectrum of activity, including, in addition to the previously mentioned ones, anti-inflammatory, antiviral, antiplatelet, antiestrogenic and anticonvulsant effects [1]. Differences in the chemical structures of individual lactones have a great influence on the scope and strength of their biological activity. Anti-cancer properties of these compounds are influenced by the presence and location of various substituents at the phenyl ring, and for the chiral lactones, also by the configurations of their stereogenic centers [2,3,4,5,6].

**Figure 1:**
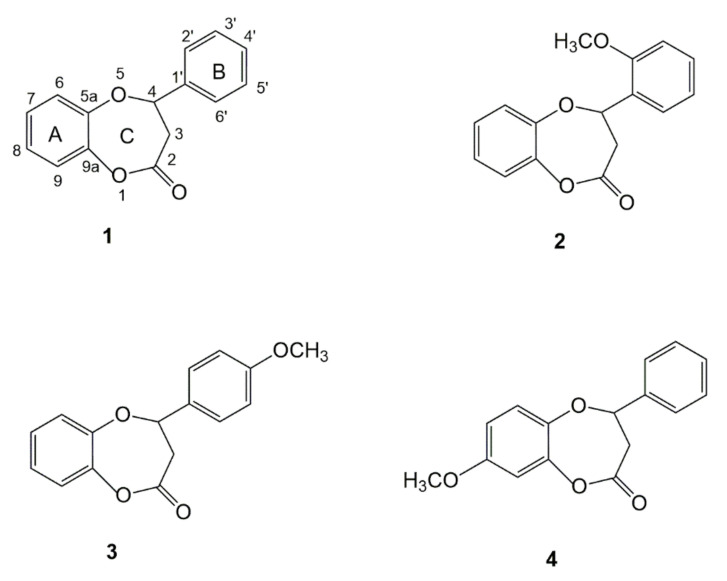
Structure of flavanone-derived γ-oxa-ε-lactone **1** and its methoxy derivatives **2**–**4**.

Among others, cytostatic activity was found for some naturally occurring seven-membered ring lactones, such as floresolide B [7]. Previous studies also showed that some flavanone-derived methoxy-substituted ε-lactones have more apoptotic activity than their parent flavanones [8]. It was also confirmed that methoxylated flavonoids easily penetrate into the cells through membranes showing increased anti-cancer properties [9]. Therefore, the aim of our research was to check the anti-tumor activity of flavanone-derived γ-oxa-ε-lactones with methoxy groups in different positions of the A and B rings. Such a panel of tested compounds allowed us to compare the strength of in vitro anti-tumor activity of different substitution patterns of the flavonoid skeleton. The subjects of the research were four compounds: parent compound **1** (lactone with unsubstituted phenyl rings) and its three derivatives (**2**, **3**, **4**) with methoxy groups in positions 2′, 4′ and 8, respectively (Figure 1). They were synthesized and characterized previously by our group as compounds with antibacterial and antifungal activity [10].

In our study, we used an interesting research model of canine lymphoma/leukemia cells. Such a model on the one hand facilitates development of new therapies for canine neoplastic diseases (apart from infectious diseases, cancer is the main cause of death in dogs), and on the other hand, the dogs are excellent animal models for studying human non-Hodgkin’s lymphomas (due to spontaneous formation and similarity of the clinical course) [11]. Research results derived from such a model can therefore be useful for both veterinary and human oncologists, paving the path for development of comparative oncology.

## 2. Results

### 2.1. Cell Viability Assay

**Figure 2:**
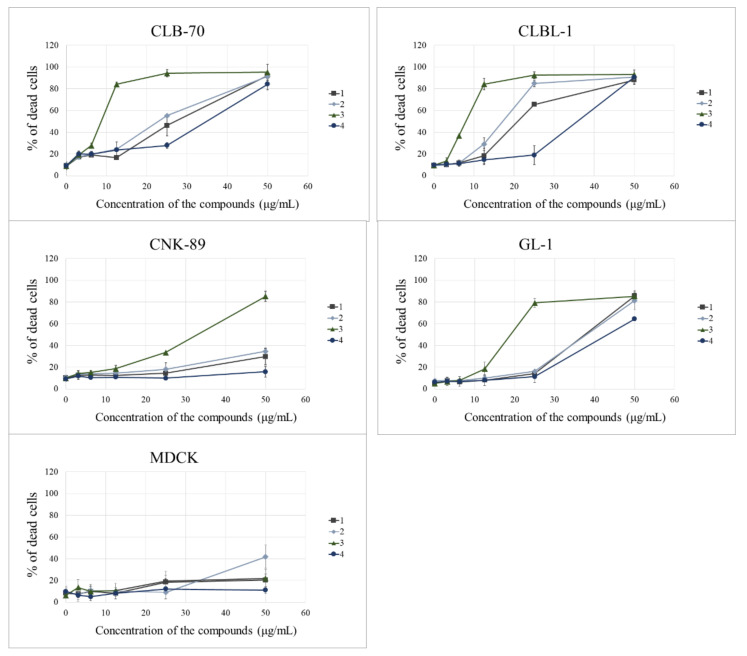
Concentration-dependent curves presenting the effects of the tested flavanone-derived γ-oxa-ε-lactones on the viability of canine cancer cell lines (CLB-70, CLBL-1, CNK-89 and GL-1) and noncancerous canine cell line (MDCK) after 48 h of incubation with different concentrations of individual lactones (3.125, 6.25, 12.5, 25 and 50 µg/mL). The values are means from three independent experiments. Last picture shows statistical differences in IC_50_ (µg/mL concentration of the tested compound that inhibits proliferation of 50% of cells) for all tested compounds in each cell line, necessary to compare how the activity of the individual derivatives differs. The results are presented as mean ± standard deviation (SD) of three separate experiments with three wells each. Results were analyzed using one-way ANOVA followed by Tukey’s multiple comparison test. Values without common letters (a, b, c) in the superscript differ statistically (*p* < 0.05). N.A.—not achieved.

The study began with checking the cytotoxic activity of the tested compounds by means of propidium iodide staining and flow cytometry analysis. All tested compounds at the concentration range up to 50 µg/mL exhibited cytotoxic activity. The differences in their strength were visible for individual derivatives (Figure 2) and individual cell lines (Table 1). At first, we compared the effects of the presence and position of the methoxy group on the strength of cytotoxic activity of the tested compounds. The modification of the parent compound **1** by an addition of methoxy substituents increased or decreased the cytotoxic activity of the new derivative, depending on the position of the group. Excluding the resistant CNK-89 cell line, the introduction of the methoxy group into position 8 of the A ring significantly lowered the activity in comparison with the parent compound, and γ-oxa-ε-lactone **4** was also less effective than other derivatives. However, the presence of the methoxy group in the B ring increased the cytotoxic activity of the resulting compounds, which was particularly noticeable in the case of lactone with the methoxy group at position 4′ (**3**) when tested on the CLB-70 cell line.

In addition, the cytotoxic effect in the noncancerous canine cell line was evaluated and it was found that in this cell line none of the compounds was able to kill 50% of the cells and only compound **2** in the highest concentration induced marked cell death in this cell line. Detailed results are shown in Figure 2.

Not only tested γ-oxa-ε-lactones differed in their activity but the cell lines also showed different sensitivity to their action. While the cell lines representing typical B-cell proliferation turned out to be highly sensitive to the anti-proliferative effect of the examined lactones, the GL-1 cell line representing B/T-cell leukemia seemed slightly less sensitive, and the cell line formed by neoplastic NK cells was clearly resistant. Within the range of concentrations tested, it was not possible to determine IC_50_ for this cell line, except for the most potent derivative—lactone **3**. Detailed differences in the sensitivity of each cell line to individual compounds are shown in Table 1.

### 2.2. Apoptosis Study

#### 2.2.1. Annexin V/PI Staining

Since the assay with propidium iodide alone showed the cytotoxic effect of the tested compounds, we decided to check the type of cell death (apoptosis or necrosis) induced by the tested compounds. To distinguish apoptosis from necrosis the cells were additionally stained with annexin V, which binds with phosphatidylserine externalized in apoptotic cells.

**Figure 3:**
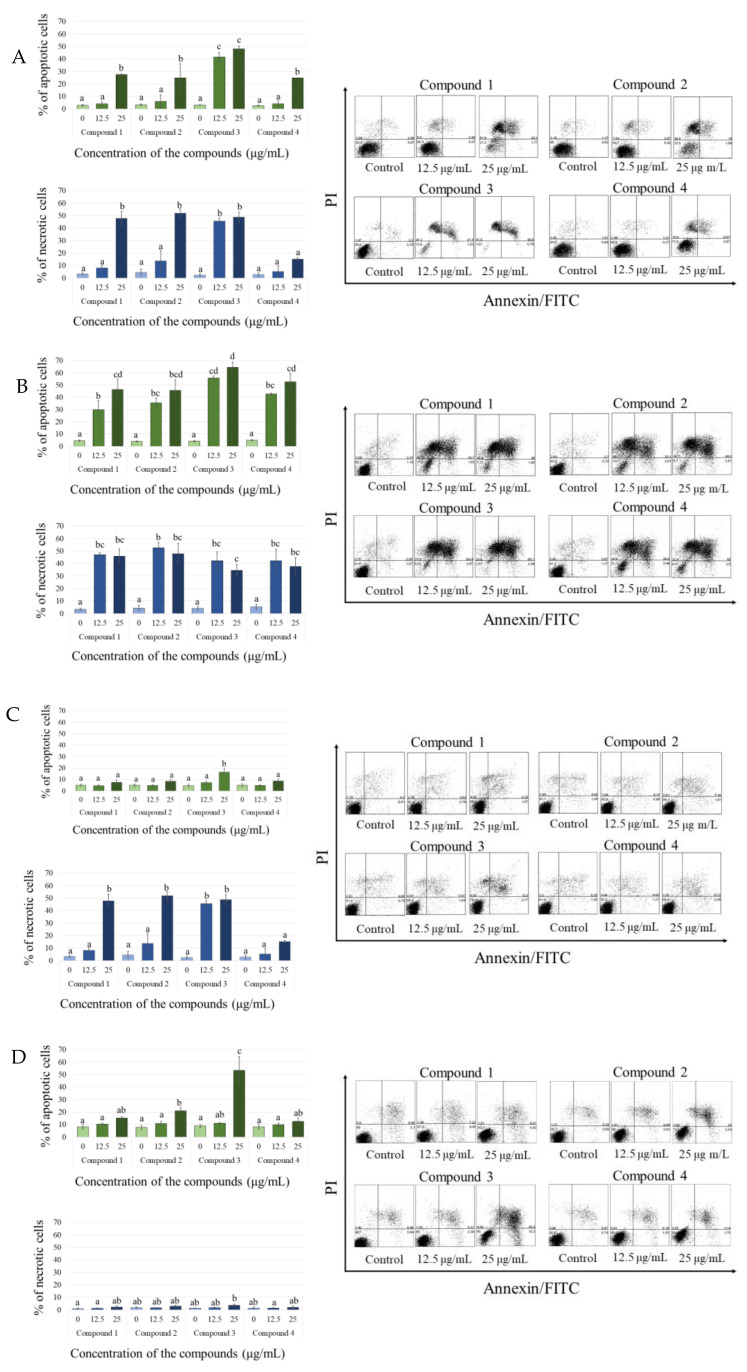
Percentage of apoptotic and necrotic cells in CLBL-1 (**A**), CLB70 (**B**), CNK89 (**C**) and GL-1 (**D**) after 48 h of incubation with the culture medium alone (0) or 12.5 and 25 µg/mL of the tested flavanone-derived γ-oxa-ε-lactones. On the right: representative dot-plots of annexin V/PI staining. The values are means of three independent experiments. Statistical differences were analyzed using one-way ANOVA followed by Tukey’s multiple comparison test. Values without common letters (a, b, c) in the superscript differ statistically (*p* < 0.05).

The experiment revealed both types of cell death ensuing from the treatment with the tested compounds. A particularly high percentage of necrotic cells was found in CLB70 and CLBL-1 cell lines, which were the most sensitive to the tested lactones. The population of necrotic cells was accompanied by the population of cells in late apoptosis also at the lowest (12.5 µg/mL) concentration of the tested substances. The strongest effect was again shown for lactone **3**, but we observed no differences in the type of cell death induced by the tested compounds. Similarly, in the two less sensitive cell lines, GL-1 and CNK-89, the populations of necrotic and late apoptotic cells were of comparable size. Detailed results along with representative dot-plots showing apoptotic and necrotic cells are presented in Figure 3.

#### 2.2.2. WB Analysis

**Figure 4:**
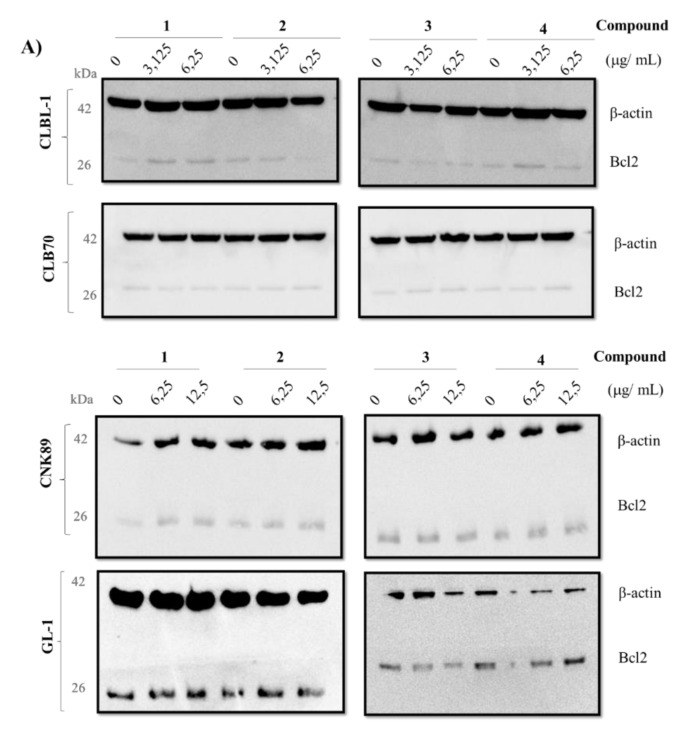

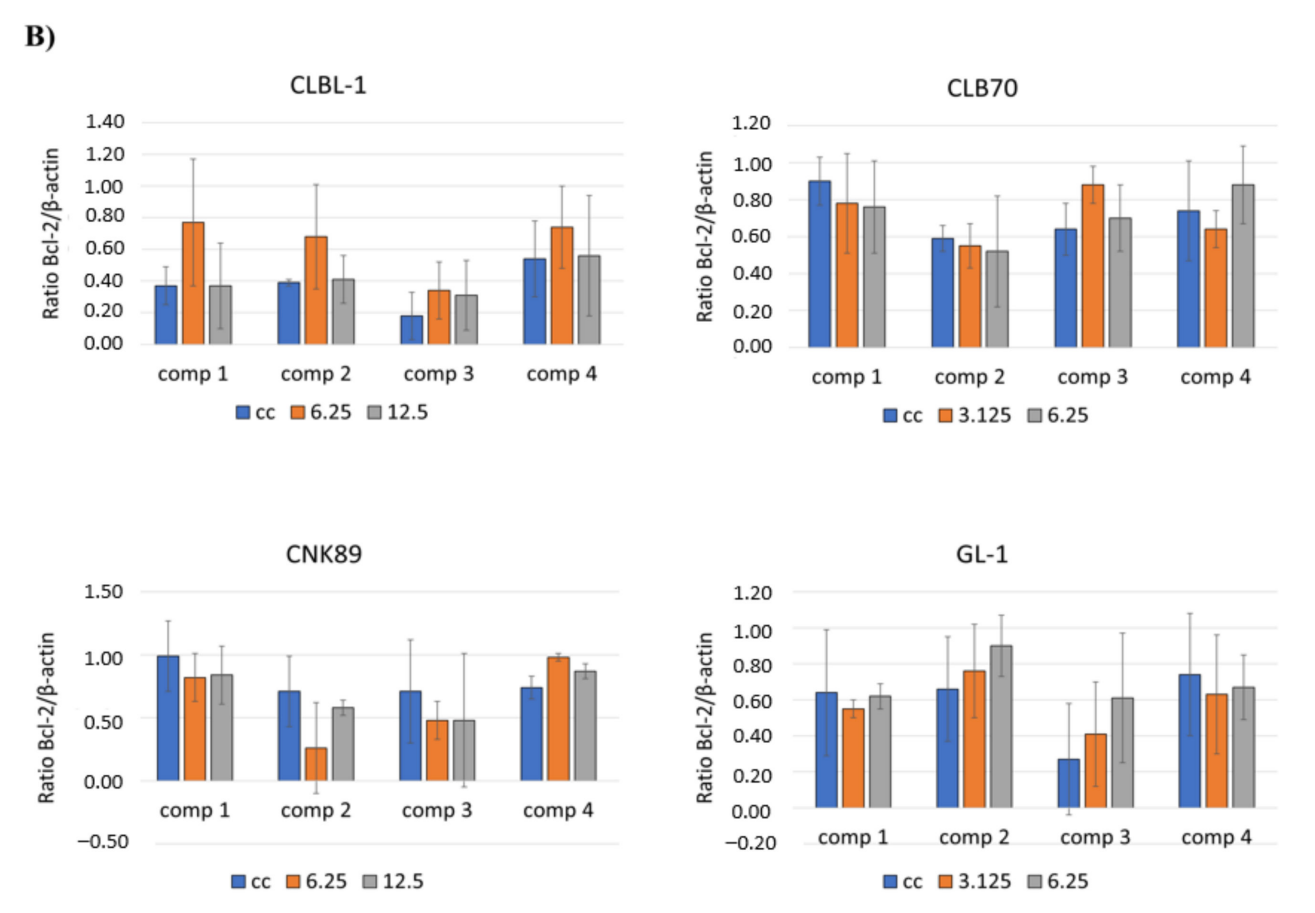
(**A**) Western blot analysis for Bcl-2 protein after 48 h of incubation with different concentrations of flavanone-derived γ-oxa-ε-lactones: 3.125 and 6.25 µg/mL for the sensitive cell lines (CLBL-1 and CLB70) and 6.25 and 12.5 µg/mL for the resistant ones (CNK-89 and GL-1). (**B**) Protein quantification. The results are presented as mean ± standard deviation (SD) of three separate experiments. Statistical differences were analyzed using one-way ANOVA followed by Tukey’s multiple comparison test. No significant differences were found between the different concentrations for the same compound.

Weak apoptosis induction was also confirmed by the Western blot, which showed no statistically significant changes in the expression level of the most important anti-apoptotic protein, i.e., Bcl-2. As the imbalance between pro- and anti-apoptotic proteins occurs only in living cells, relatively low concentrations of tested lactones were used in the study: 3.125 and 6.25 µg/mL for the sensitive cell lines (CLBL-1 and CLB70) and 6.25 and 12.5 µg/mL for the resistant ones (CNK-89 and GL-1). Detailed results are shown in Figure 4.

### 2.3. Study of Synergistic Action of Flavanone-Derived γ-Oxa-ε-Lactones and Glucocorticoids

**Figure 5:**
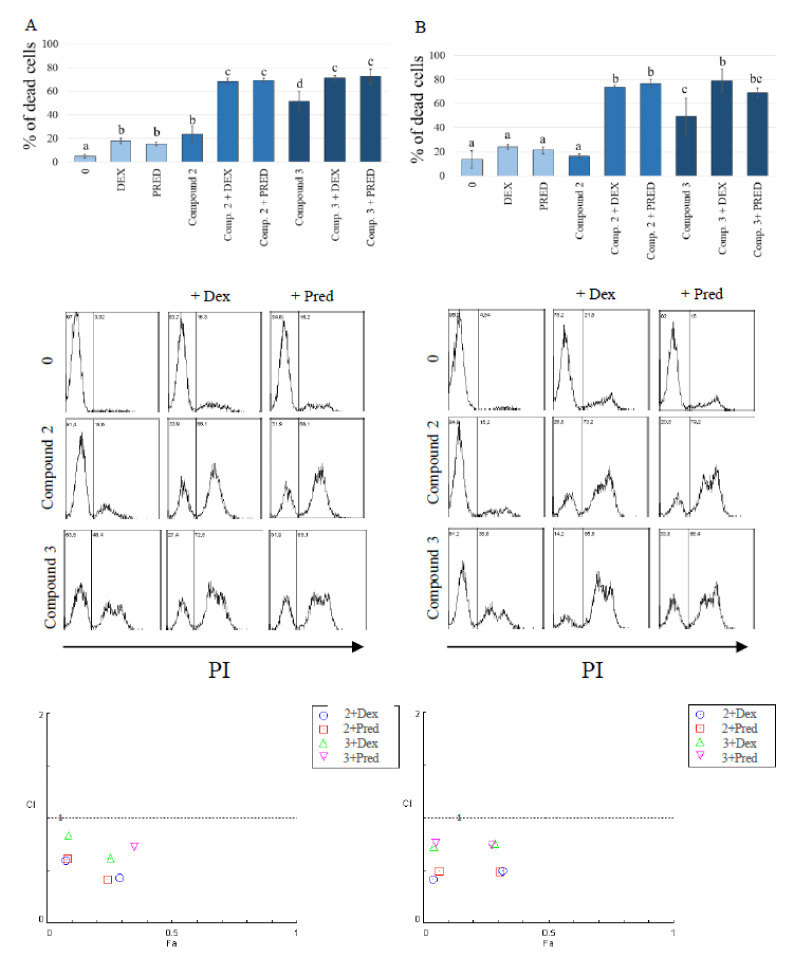
Synergistic effect of flavanone-derived γ-oxa-ε-lactones (3.125 µg/mL) combined with dexamethasone (Dex) or prednisolone (Pred) (2.5 µg/mL) in CLBL-1 (**A**) and CLB70 (**B**) cell lines. The values are means of two independent experiments. Statistical differences were analyzed using one-way ANOVA followed by Tukey’s multiple comparison test. Values without common letters (a, b, c) in the superscript differ statistically (*p* < 0.05). Combination index (CI) versus fraction affected (Fa) for combination of lactone **2** and **3** with dexamethasone and prednisolone was calculated using the Chou–Talalay method. Synergy was defined by CI values <1.

**Figure 6:**
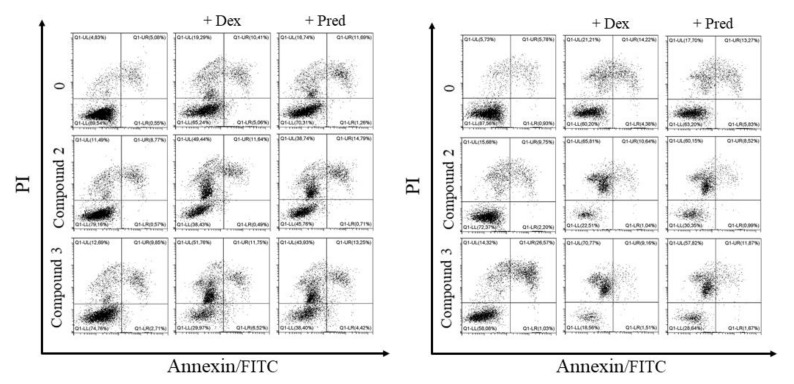
Synergistic effect of flavanone-derived γ-oxa-ε-lactones (3.125 µg/mL) combined with dexamethasone (Dex) or prednisolone (Pred) (2.5 µg/mL) in CLBL-1 (**left**) and CLB70 (**right**) in annexin V and propidium iodide double staining. Visible strong induction of necrotic instead of apoptotic cell death after the use of the lactone and corticoid combination.

As our research demonstrated the anti-tumor potential of the tested compounds, we decided to check whether the obtained derivatives could be used in the treatment of lymphomas/leukemias in dogs. To this end, we resolved to verify whether the tested compounds act synergistically with common anti-cancer drugs used in canine therapy. As each therapeutic regimen used in the treatment of hematopoietic neoplasms in dogs contains glucocorticosteroids, we investigated the lactones in combinations with dexamethasone and prednisolone. The two most potent methoxy substituted lactones, **2** and **3**, were selected for these tests with two cell lines (CLBL-1 and CLB70) most sensitive to the lactone action. The experiments clearly showed that the combination of glucocorticosteroids (both dexamethasone and prednisolone) with the flavonoid derivatives induced stronger cell death than either of the substances alone. Synergy evaluation confirmed the synergistic effect for the combinations of dexamethasone and prednisolone with lactones **2** and **3** in both cell lines (synergistic effect was identified by combination index (CI) values less than 1). The effect was most pronounced at low concentrations of the tested lactones (3.125 µg/mL) in combination with 2.5 µg/mL of steroid drugs. In these concentrations, in the CLB70 cell line, CI was about 0.4 for combinations of lactone **2** and both glucocorticosteroids while for lactone **3** it was about 0.62 and 0.76 for dexamethasone and prednisolone, respectively. In the CLBL-1 cell line, the CI for lactone **2** in combination with dexamethasone or prednisolone was about 0.5, while for lactone **3** it was about 0.75. At the higher concentration the lactones were so effective in inducing cell death that the effect of their combination with steroids could not be observed. Detailed results are shown in Figure 5. Knowing that the combination of compounds used causes a stronger cytotoxic effect than each of the compounds alone, we also decided to check whether the type of induced cell death had changed. A study using annexin V (apoptotic cells) and propidium iodide (dead/necrotic cells) double staining showed no difference in the type of induced cell death. As with the single compounds, combinations of tested lactones with glucocorticosteroids mainly induced necrotic cell death (Figure 6).

## 3. Discussion

Despite years of intensive research into the causes and treatment of cancer, it still remains a huge and unsolved problem for mankind. According to the data provided by the World Health Organization (WHO) in 2018, the global annual number of new cancer cases is estimated at about 18.1 million. The same source states the specific cancer-related mortality at about 10 million yearly. Moreover, the WHO estimates that the global cancer incidence could increase by more than 63% in 2040 as compared with 2018 [12]. Such a high incidence and mortality rate due to cancer necessitates intensive studies on the search for novel therapeutic agents.

The search for new compounds with potential anti-cancer activity among agents of natural origin is still a popular field of research. In fact, the treatment of cancer has started with the use of natural substances. An example here could be basic cytostatic drugs such as irinotecan, vincristine, etoposide and paclitaxel sourced from plants, actinomycin D and mitomycin C produced by microorganisms or marine-derived bleomycin [13]. Flavonoids, plant secondary metabolites with specific phenolic structures, are also a good example. These bioactive compounds can be found in the flowers of different medicinal plants but also in vegetables, fruit, roots or bark. Moreover, they are ingredients of popular drinks such as tea or wine [14]. Due to their numerous biological activities, flavonoids have an established position in the world of medicine, especially in the treatment of chronic diseases such as cancer. In relation to cancer treatment, the most important action of these compounds is primarily their anti-proliferative activity confirmed by numerous studies, but also the ability to induce apoptosis, autophagy, necrosis, cell cycle arrest, senescence, the impairment of cell migration, invasion, tumor angiogenesis and the reduction of multidrug resistance in tumor cells [15]. Another very interesting group of natural compounds with potential applications in oncology are lactones, i.e., secondary metabolites produced by plants, bacteria, fungi, marine sponges and other organisms. It is estimated that more than 3000 γ-lactones occur in nature. The majority of γ-lactones exhibit fascinating biological activities such as herbicidal, insecticidal and antifouling, and some of them also have anti-cancer properties [16]. Lactones are used as aroma substances in the cosmetics and perfume industries [17] and as flavorings in the food industry [18].

Considering the above, we decided to combine our knowledge of the anti-cancer properties of flavonoids and natural compounds containing lactone moiety to create new compounds with the advantages of both these groups. Since poor bioavailability penetration into the cells through membranes may significantly limit the activity of the flavanone-derived lactones, we decided to modify the structure of our compounds by synthesis of methoxy derivatives. We also resolved to determine if the location of the methoxy group in the tested compounds would affect their activity.

Our previous research demonstrated that introduction of lactone function increased anti-microbial activity of a tested compound as compared with its flavonoid precursors [10]. Studies by another group revealed a similar trend for anti-tumor activity by indicating that a conversion of naringenin and hesperetin in a heterogeneous catalytic Baeyer–Villiger reaction into lactones increased the pro-apoptotic activity of the tested compounds [8]. In this study, we did not compare the anti-tumor activity of the original flavonoids with their lactone derivatives, but we proved that the conversion of flavanones into lactones yields compounds with high anti-cancer activity in vitro.

Further observations concerned the comparison of the presence and location of the methoxy group on the anti-cancer activity of the tested lactones. Although substitution of the aromatic ring with the methoxy group should positively affect the ability of the compound to enter the cell [9,19], we found that the presence of the methoxy group in the A ring at C-8 yields a derivative (**4**) with lower activity than that of the parent compound **1** (Figure 2, Table 1). It is interesting, as according to the literature, that the flavonoid *O*-methylation contributes to increased biological activity often associated with ring A polymethoxylation. Excellent examples are nobiletin and tangeretin offering the greatest proliferation inhibition among all Ougan flavonoids, which also suggests the importance of C-8 substituents for antiproliferative activity of flavonoids [20]. At the same time, our study clearly indicated that substitution of the methoxy group on the B ring enhanced the cytotoxic activity of the obtained derivatives. Lactone **2**, substituted with the methoxy group at the 4′ position, was the compound with the strongest anti-cancer activity, about 2- or 3-fold stronger than that of the parent compound, depending on the cell line used. A structural isomer of lactone **3**, namely 2′-methoxy derivative **2**, showed lower cytotoxic potency, as illustrated in Table 1 by IC_50_ values for each derivative. Both compounds were more effective towards CLBL-1 and CLB70 cell lines (representing B-cell lymphoma and chronic type B leukemia, respectively) [21,22] than towards the rarer and more difficult-to-treat types of hematopoietic cancer in dogs—acute leukemia of not clearly defined phenotype (B/T) [22,23], or NK cell lymphoma [24]. The remaining tests confirmed that the difference between these isomers concerns only the strength of their action and not the way in which they kill cancer cells. The presence of the methoxy group in the 4′ position of the B ring increased potency of the compound but did not promote apoptosis over necrosis in any cell line or derivative. Such a conclusion can be drawn from the experiments with annexin V and propidium iodide, which demonstrated a comparable ratio of apoptotic to necrotic cells after their treatment with each of the tested lactones (Figure 3). In addition, the Western blot assay, used for visualizing changes in the expression of the Bcl-2 protein involved in the apoptosis, did not confirm a more pronounced effect of 2′-methoxylactone **2** and/or 4′-methoxylactone **3** on the balance between pro- and anti-apoptotic proteins in the cell that would facilitate the course of apoptosis (Figure 4). It can therefore be concluded that the substitution site of the methoxy group does not affect the mechanism of the cytotoxic action of the tested flavanone-derived lactones.

At the final stage of the study, we assessed the potential use and role of the obtained lactones in the treatment of hematopoietic cancers. A combination treatment strategy, which serves as an important direction for the development of natural products in cancer therapy, seems an interesting research direction. Adjuvant treatment with natural products supplementing current treatment regimens could be beneficial in multiple aspects, including mitigation of adverse effects, overcoming drug resistance and improving therapeutic response. The idea of a combination therapy with flavonoids is already being researched. Firstly, numerous studies confirmed that flavonoids show better anti-tumor activity when used in complex preparations containing a mixture of several compounds from this group [25,26,27,28,29,30,31]. Such reports mainly focused on the health-promoting role of food rich in flavonoids. Secondly, flavonoids were noted to act synergistically with classic anti-cancer drugs. Examples involve a synergistic effect of flavonoids from *Artocarpus heterophyllus* heartwoods on the anti-cancer activity of cisplatin against h460 and mcf-7 cell lines [32], synergistic pro-apoptotic effects of epicatechin in combination with cisplatin in renal tubular carcinoma [33], or quercetin reversing docetaxel resistance in prostate cancer via androgen receptor and PI3K/Akt signaling pathways [34]. More and more reports also suggest the use of flavonoids with the latest, molecularly targeted therapies, such as a combination with the kinase inhibitor sorafenib [35] or immune checkpoint inhibitors [36].

To initially assess the usefulness of the lactones in the adjuvant therapy of lymphomas and leukemias, we examined their synergistic action with basic drugs used in the treatment of hematopoietic neoplasms, i.e., glucocorticosteroids. This preliminary study, involving two canine cancer cell lines, confirmed the synergistic effect of both dexamethasone and prednisolone with the two lactones with the highest cytotoxic activity, **2** and **3**. We found that non-toxic concentrations of both glucocorticosteroids (2.5 µg/mL) markedly potentiated the killing effect of these derivatives. The effect of the combination was so strong that cell viability could only be assessed at the lowest concentration of lactones used in the study (3.125 µg/mL). The scope and strength of the cytotoxic effect of the proposed combination as well as its mechanism of action require further research but we established the obtained compounds as useful adjuvants in standard therapeutic protocols and possibly as an interesting alternative for classic chemotherapy.

## 4. Materials and Methods

### 4.1. Compounds

The study investigated four flavanone-derived γ-oxa-ε-lactones (**1**–**4**) obtained in a three-step synthesis from corresponding flavanones as described earlier [10]. The structures of the tested lactones are shown in Figure 1.

### 4.2. Compound Preparation

All tested compounds were dissolved in dimethyl sulfoxide (DMSO) to prepare 10 mg/mL stock solution. The stock solutions were stored at room temperature. Tested dilutions were obtained by dissolving the stock solution in a complete culture medium.

### 4.3. Cell Lines and Cell Culture

The study involved a panel of canine cancer cell lines: CLBL-1 (B-cell lymphoma), GL-1 (B/T-cell leukemia), CLB70 (B-cell chronic lymphocytic leukemia) and CNK-89 (NK-cell lymphoma), and a normal canine cell line (MDCK). CLBL-1 was obtained from Barbara C. Ruetgen from the Institute of Immunology, Department of Pathobiology, University of Veterinary Medicine, Vienna, Austria [22]; GL-1 from Yasuhito Fujino and Hajime Tsujimoto of the University of Tokyo, Department of Veterinary Internal Medicine [23]; while CLB70 [21] and CNK-89 [24] were established in our laboratory. The MDCK cell line was purchased from Sigma Aldrich (Steinheim, Germany).

The cell lines were maintained in RPMI 1640 (CLBL-1, GL-1 and MDCK) (Institute of Immunology and Experimental Therapy, Polish Academy of Sciences, Wrocław, Poland) or Advanced RPMI (Gibco, Grand Island, NY, USA) (CLB70 and CNK-89) culture medium supplemented with 2 mM L-glutamine (Sigma Aldrich, Steinheim, Germany), 100 U/mL penicillin, 100 μg/mL streptomycin (Sigma Aldrich, Steinheim, Germany) and 10–20% heat-inactivated fetal bovine serum—FBS (Gibco, Grand Island, NY, USA).

### 4.4. Cell Viability Assay

To evaluate and compare the effects exerted by γ-oxa-ε-lactones 1–4 on the cultures of canine lymphoma (CLBL-1, CNK-89) and leukemia (GL-1, CLB70) cell lines, the cells were treated with increasing concentrations of each compound (3.125, 6.25, 12.5, 25 and 50 µg/mL) for 48 h. This incubation time was chosen after preliminary studies showed that 48 h of treatment of the cells with the tested compounds allows both the evaluation of the cytotoxic effect and the observation of late signs of apoptosis. The cells were seeded at a concentration of 1 × 10^4^ cells per well in a 96-well plate (TPP, Trasadingen, Switzerland). The cells were incubated in the medium alone or in the medium containing either the vehicle control or increasing concentration of the tested substance for 48 h. After the incubation, the cells were harvested and washed twice in PBS, transferred into cytometric tubes and stained with propidium iodide (PI, final concentration 1 μg/mL). Flow cytometry analysis was performed immediately, using a flow cytometer FACS Calibur (Becton Dickinson, Biosciences, San Jose, CA, USA). CellQuest 3.lf. software (Becton Dickinson, San Jose, CA, USA) was used for data analysis on the basis of histograms of FL2-H showing the population of alive (PI negative) and dead (PI positive) cells. The values presented as means with standard deviations were obtained from three independent experiments and shown as a concentration-dependent curve. IC_50_ for the tested compounds was calculated as a mean concentration inhibiting cell viability by 50% in three independent experiments.

The method of staining cells with propidium iodide was also used to evaluate a synergistic effect of the tested derivatives with glucocorticosteroids. For this study, the two most potent derivatives **2** and **3,** and two cell lines (CLBL-1 and CLB70) most sensitive to the activity of the tested lactones, were selected. Prednisolone and dexamethasone, frequently used in the treatment of lymphomas and leukemias in dogs, were tested as glucocorticoid compounds. The cells were incubated with individual compounds or their combinations for 48 h. The range of the tested concentrations for a single treatment was 3.125, 6.25 and 12.5 µg/mL for lactones and 2.5, 5 and 10 µg/mL for glucocorticoids. Dexamethasone and prednisolone concentrations of 2.5 µg/mL were chosen to evaluate the synergistic effect, and these drugs were added together with the lactones in the entire range of concentrations tested (3.125, 6.25 and 12.5 µg/mL). Synergy was estimated using CompuSyn software and the Chou-Talalay method [37]. The synergistic effect was identified by combination index (CI) values less than **1**.

### 4.5. Western Blotting

For Western blot analysis the cells were seeded in a total of 5 × 10^6^ cells per 25 cm^2^ cell culture flasks and the compounds were added at two selected concentrations (3.125 and 6.25 µg/mL for the resistant cell lines and 6.25 and 12.5 µg/mL for the sensitive cell lines). After 48 h incubation the cells were harvested, rinsed with cold PBS, suspended in a lysis buffer (50 mM Tris–HCl pH 7.5, 100 mM NaCl, 1% NP-40 and protease inhibitors set) and incubated for 20 min on ice. The suspensions were centrifuged at 10,000 rpm at 4 °C for 12 min. Then, a sodium dodecyl sulfate (SDS) sample buffer was added to clear supernatants, and the samples were boiled at 95 °C for 5 min and subjected to SDS-PAGE on 12% gel. The resolved proteins were transferred to a PVDF membrane (Millipore, Billerica, MA, USA), using Semidry Transfer Cell (Bio-Rad, Hercules, CA, USA). After the transfer, the membrane was blocked for 1 h with 3% bovine serum albumin (BSA) in TBS at room temperature, and then incubated with primary antibody (dilution 1:1000) (Santa Cruz Biotechnology, Santa Cruz, CA, USA) at 4 °C overnight, followed by secondary horseradish peroxidase-labeled antibody (Dako, Denmark) for 1 h at room temperature. The bound antibodies were visualized using ChemiDoc Touch Instruments (BioRad, Hercules, CA, USA). The anti-β actin (C-4) and anti-Bcl-2 (C-2) antibodies from Santa Cruz Biotechnology (Santa Cruz, CA, USA) were used in this study. For Bcl-2 expression quantification, Western blot normalization using a single protein (housekeeping protein, β-actin) was performed using Image LabTM software (version 5.2.1; BioRad).

### 4.6. Flow Cytometry Apoptosis Assays

Early apoptotic events were detected by means of annexin V FITC/PI staining. Briefly, the cells were treated with the tested compounds at 12.5 and 25 µg/mL for 48 h. The concentrations of the compounds were the same as for determination of cell viability. After harvesting the cells and washing them twice with cold PBS, they were suspended in a binding buffer and stained with annexin V-FITC for 10 min at room temperature or PI (final PI concentration, 1 µg/mL). Immediately after, the incubation flow cytometric analysis was performed using a FACS Calibur analyzer (Becton Dickinson, Biosciences, San Jose, CA, USA). CellQuest 3.lf. software was used for data analysis.

### 4.7. Statistical Analysis

All data are shown as means with standard deviations (SD). Statistical differences were analyzed using one-way ANOVA followed by Tukey’s multiple comparison test. Statistical analysis was performed with STATISTICA version 13.3 software (TIBCO Software Inc., Palo Alto, CA, USA). The results were considered significant at *p* < 0.05.

## 5. Conclusions

The tested flavanone-derived lactones show anti-cancer activity in vitro. Depending on its location, the presence of the methoxy group in the molecules can both increase or decrease their cytotoxicity in comparison with the parent compound **1**. The most potent lactone was obtained by the introduction of the methoxy group at position 4′ of the B ring (compound **3**), whereas the presence of this group at C-8 in the A ring resulted in its lower activity. The substitution pattern of methoxy-substituted flavanone-derived lactones did not affect the mechanism of the cytotoxic action of the investigated compounds but influenced the strength of their anti-cancer activity. In vitro examination of the potential synergistic anti-tumor activity of the tested lactones with glucocorticoids showed that this combination was more potent than either of the compounds alone. This may indicate a potential for the application of the lactones in the adjuvant treatment of hematopoietic neoplasms in the future. However, further studies on the mechanism of action and possible in vivo use of the obtained flavanone-derived lactones are required.

## Figures and Tables

**Table 1:** IC_50_ (µg/mL concentration of the tested compound that inhibits proliferation of 50% of cells) for all tested compounds and all cell lines used in the study, obtained by PI staining after 48 h treatment. The results are presented as mean ± standard deviation (SD) of three separate experiments with three wells each. Statistical differences were analyzed using one-way ANOVA followed by Tukey’s multiple comparison test. Values without common letters (a, b, c) in the superscript differ statistically (*p* < 0.05). The table shows differences in the strength of action of each compound on selected cell lines. N.A.—not achieved. N.I.—not investigated.

Compound/Cell Line	CLB-70	CLBL-1	CNK-89	GL-1
1	26.13 ± 3.55 ^a^	19.96 ± 1.05 ^b^	N.A.	37.52 ± 1.27 ^c^
2	23.24 ± 2.63 ^a^	17.14 ± 0.59 ^a^	N.A.	36.41 ± 4.41 ^b^
3	8.25 ± 1.14 ^a^	7.99 ± 0.19 ^a^	31.52 ± 3.18 ^b^	18.95 ± 0.47 ^c^
4	31.94 ± 7.88 ^a^	31.49 ± 4.21 ^a^	N.A.	42.81 ± 0.07 ^a^
Etoposide	14.33 ± 2.12	0.02 ± 0.00	N.I.	4.43 ± 1.08

## Data Availability

The data presented in this study are available on request from the corresponding author.

## Sample Availability

No available.
